# Efficacy and Safety of Static Stretching and Daily Walking on the Cardio-Ankle Vascular Index in a Patient With Type 2 Diabetes Mellitus, Proliferative Retinopathy, and Lower-Extremity Peripheral Arterial Disease: A Case Report

**DOI:** 10.7759/cureus.74769

**Published:** 2024-11-29

**Authors:** Ryota Shinomiya, Hinata Fukuike, Masaaki Nakajima

**Affiliations:** 1 Rehabilitation Department, Tokushima Kensei Hospital, Tokushima, JPN; 2 Research Institute of Health and Welfare, Kibi International University, Takahashi, JPN; 3 Human Sciences, Kibi International University, Takahashi, JPN

**Keywords:** cardio-ankle vascular index, diabetes mellitus, peripheral arterial disease, proliferative retinopathy, static stretching, walking

## Abstract

Proliferative diabetic retinopathy (PDR) and peripheral arterial disease (PAD) of the lower extremities are serious complications of type 2 diabetes mellitus (T2DM). Aerobic exercise has been shown to be primarily effective for glycemic control and gait disturbance owing to PAD. However, the safety and efficacy of exercise therapy in patients with PDR remain unclear. The purpose of this case report was to demonstrate the short-term effects of static stretching (SS) and daily walking over 10,000 steps on patients with T2DM presenting with PDR and PAD. The intervention consisted of 40 minutes of other-movement SS and in-hospital walking during a two-week hospitalization phase and 20-30 minutes of automatic SS and outdoor walking during a two-week home phase post-discharge. The walking conditions consisted of 10,000 steps/day, and the rate of perceived exertion was 11-12 on the Borg scale. Outcomes included the presence of a new intraocular hemorrhage and changes in intraocular pressure (IOP), blood pressure, fasting blood glucose level, biochemical parameters, cardio-ankle vascular index (CAVI), and ankle-brachial index. No new intraocular hemorrhage or increased IOP was observed during the intervention period, and blood pressure, glycemic control, and CAVI parameters improved. The results of the intervention in this case suggest that the combination of SS and walking exercises may be safe and effective.

## Introduction

Globally, the number of people having diabetes mellitus (DM) is 537 million. DM affects 1 in 10 adults (10.5%), caused 6.7 million deaths in 2021, and kills 1 person every 5 seconds worldwide. DM is a major healthcare economic problem, with a healthcare cost burden of Japanese Yen 110 trillion ($966 billion) in 2021 [1]. The medical cost burden of DM increased by 316% over the past 15 years. Type 2 DM (T2DM) accounts for >90% of DM cases worldwide. T2DM develops when a relative insulin deficiency occurs owing to a genetic predisposition to reduced insulin secretory capacity, combined with environmental predisposition to insulin resistance caused by a high-calorie and high-fat diet, lack of exercise, and other lifestyle factors [2]. T2DM is a chronic disease with increasing prevalence worldwide and is associated with numerous complications. Proliferative diabetic retinopathy (PDR) and peripheral arterial disease (PAD) of the lower extremities are serious complications of T2DM. PAD affects approximately 236 million people worldwide [3]. It causes pain and intermittent claudication owing to occlusion or stenosis of peripheral blood vessels, resulting in an accelerated loss of mobility [4]. Patients with PAD have a higher prevalence of atherosclerosis in coronary, carotid, and renal arteries than those without [5] and an approximately two-fold increase in mortality [6]. DM is a strong risk factor for PAD [7], and patients with DM and PAD have a higher risk of lower-limb amputation and mortality [8]. Therefore, it is important to improve glycemic control to prevent arteriosclerosis in patients with PAD. Walking exercises are recommended as conservative therapy for DM complications. Exercise therapy, primarily walking, is an effective intervention as it has been reported to improve glycemic control [9], improve arterial stiffness [10], and improve walking ability [11]. However, when PDR is present, aggressive exercise is not recommended because of neovascularization, which causes the formation of fragile new vessels in the retina and the risk of hemorrhage and retinal detachment caused by a sudden increase in blood pressure [12]. Intervention with exercise therapy is risky, especially high-intensity exercise, which can cause an increase in intraocular pressure (IOP) [13]. Therefore, static stretching (SS) and daily walking, which are classified as relatively mild intensity exercises of 2.5-3 metabolic equivalents (METs) [14], may be safer yet effective. However, there have been no reports of the use of these interventions in patients with T2DM with PDR and PAD. In this case report, we evaluated the effects of SS and active daily walking over 10,000 steps/day on glycemic control and arterial stiffness, as well as their safety in T2DM patients with PDR and PAD.

## Case presentation

Patient information

This case report is of a man in his 60s (height: 162.4 cm, weight: 59.2 kg, and body mass index: 22.4 kg/m^2^) who was admitted to Tokushima Kensei Hospital for two weeks in 2022 for glycemic control and PDR treatment (photocoagulation). He had T2DM for 10 years, and its complications included asymptomatic neuropathy, PDR, PAD (Fontaine classification grade I), stage 3 nephropathy, hypertension, and dyslipidemia. On admission, his HbA1c level was 13.1% and fasting blood glucose (FBG) level was 182 mg/dL, thus indicating poor glycemic control. The cardio-ankle vascular index (CAVI) was 9.2 on the right and 8.8 on the left, and the atherosclerotic carotid artery echocardiogram (for maximum carotid intima-media thickness in the common carotid artery) showed levels of 2.1 mm on the right and 1.8 mm on the left, indicating progressive atherosclerosis. The coefficient of variation of R-R intervals was 2.59%, and there were no autonomic abnormalities. The patient had no exercise routine prior to admission; his leg circumference was 34 cm bilaterally, the gastrocnemius manual muscle testing was level 5 bilaterally, and his comfortable walking speed was 1.3 m/s. Although the range of motion at the joints was reduced to 5° bilaterally for ankle dorsiflexion and 55° on the right and 50° on the left for straight leg raising, he did not face any problems with the exercise therapy and exhibited good understanding of the exercise instructions. The following medications were prescribed for hyperglycemia: linagliptin 5 mg (one tablet), voglibose 0.2 mg (two tablets), and repaglinide 0.25 mg (one tablet), and the prescription remained the same before and during the intervention. Calorie intake was 1,800 kcal/day (kidney diet: 60 g protein, 6 g salt) during hospitalization and 1,770 kcal/day post-discharge, which was similar. Before hospitalization, he used alcohol and consumed five 500-mL bottles of shochu (distilled spirits) a day. After discharge from the hospital, he had difficulty abstaining from alcohol but reduced the consumption to three 350-mL bottles of soju (distilled spirits) with a sugar-free label per day.

The purpose and objectives of this report and the protection of personal information were explained to the patient in writing and orally, and informed consent was obtained.

Intervention

**Figure 1:**
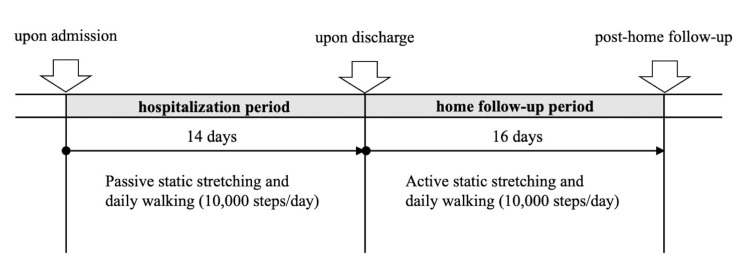
Intervention protocol

Intervention task: SS and daily walking were performed for one month during the two-week hospitalization period and two weeks post-discharge. The intervention protocol is illustrated in Figure 1.

H*ospitalization Period (Two Weeks From the Date of Admission to the Date of Discharge)*

**Figure 2:**
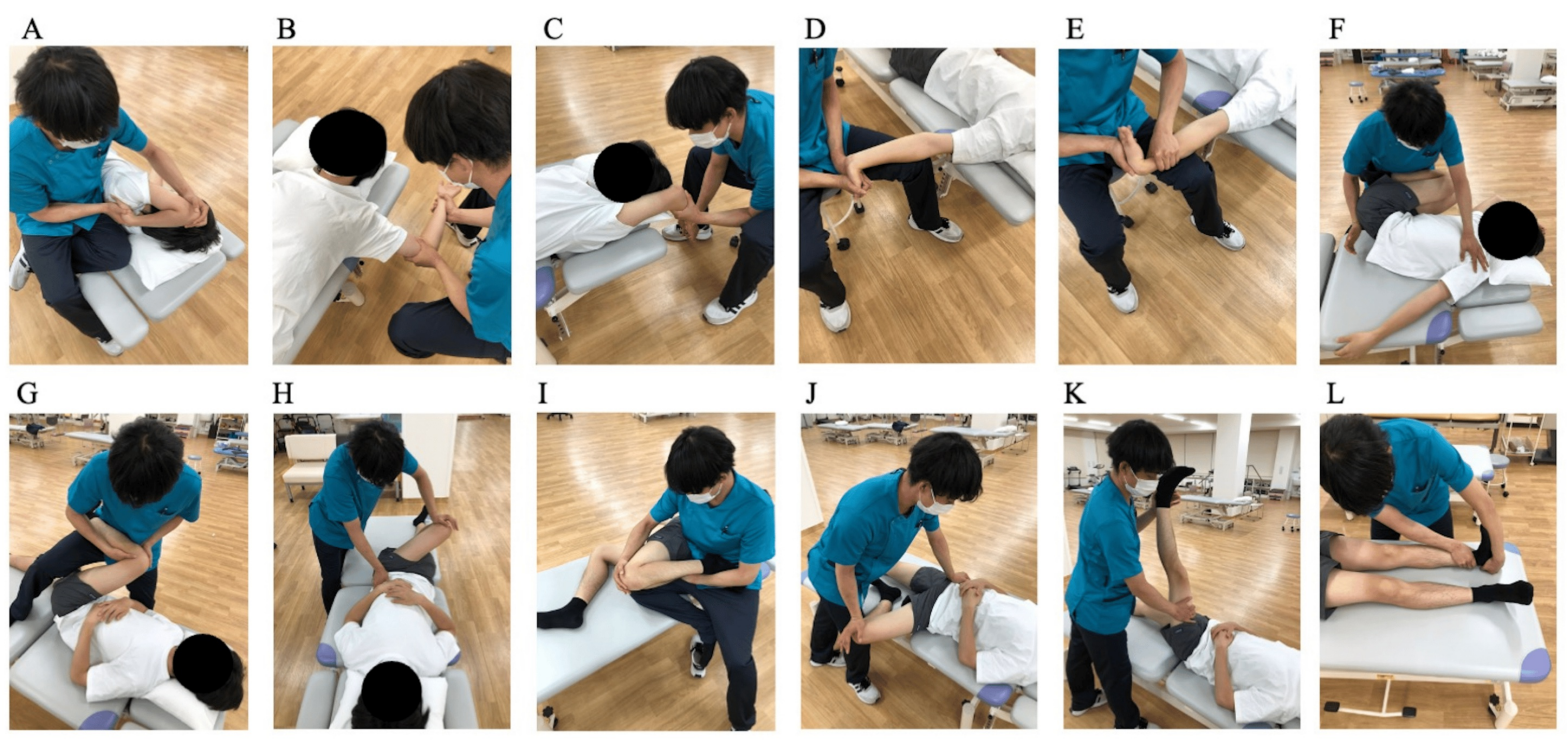
Passive static stretching This figure was originally created by the authors. (A) Posterior upper arm. (B) Chest. (C) Upper back. (D) Anterior wrist. (E) Posterior wrist. (F) Lower back. (G) Buttocks. (H) Lateral hip. (I) Anterior thigh. (J) Inner thigh. (K) Posterior thigh. (L) Calf.

Passive SS was performed for 40 min/day on the major muscle groups of the extremities, with three sets of 20 seconds of stretch and 10 seconds of relaxation for each muscle. The intensity was defined as “the maximum range of motion at which the patient felt a stretch but did not experience pain” (Figure 2). Passive SS was performed by a well-trained physical therapist, with all sessions consistently conducted by the same therapist.

The patient was instructed to walk daily at a comfortable rate of 10,000 steps/day at a subjective rate of perceived exertion of 11 to 12 on the Borg scale in the hospital. The number of steps taken was measured using an activity meter (Lifecoda GS, Suzuken Co., Nagoya, Japan).

At discharge, the patient was instructed to perform active SS for the major muscle groups of the extremities and to walk 10,000 steps/day daily until the next visit. He was given an exercise checklist and instructed to perform active SS and record the number of steps taken.

Home Follow-up Period (16 Days From the Day Post-Discharge)

**Figure 3:**
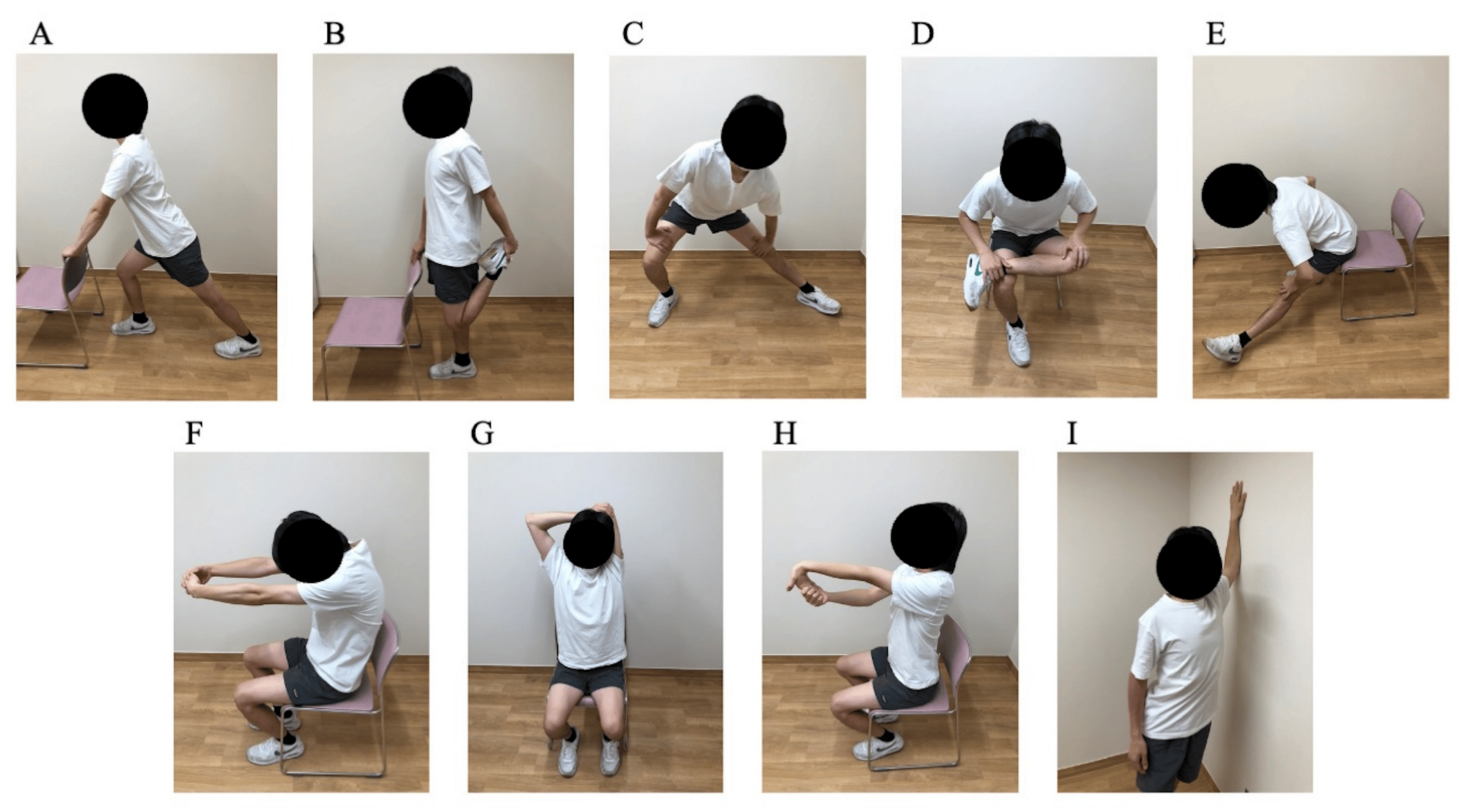
Active static stretching The figure was created by the authors based on the method described by Yamada et al. [15]. (A) Calf. (B) Anterior thigh. (C) Inner thigh. (D) Buttocks. (E) Posterior thigh. (F) Upper back. (G) Posterior upper arm. (H) Anterior wrist. (I) Chest.

Active SS for the major muscle groups of the extremities was performed for 20-30 min/day (Figure 3). Active SS consisted of three sets of 20 seconds of extension and 10 seconds of relaxation for each muscle. This intensity was similar to that of the other SS. The patient was instructed not to hold his breath during automatic SS, and an SS method that did not cause a heads-down was selected. Following the hospitalization period, the patient was fitted with an activity meter and instructed to walk outdoors at a rate of 10,000 steps/day.

Assessment Task

FBG and biochemical parameters were extracted from electronic medical records as metabolism-related indicators. CAVI and ankle-brachial index (ABI) were measured as arteriosclerosis-related indices. Intraocular hemorrhage and IOP were assessed as effects on PDR. For FBG, systolic blood pressure (SBP), and diastolic blood pressure (DBP), the slope of the regression line was determined from the daily data. Biochemical parameters were assessed at two time points: on admission and after home follow-up. CAVI and ABI were assessed at three time points: on admission, at discharge, and after home follow-up.

Outcome of the intervention

**Figure 4:**
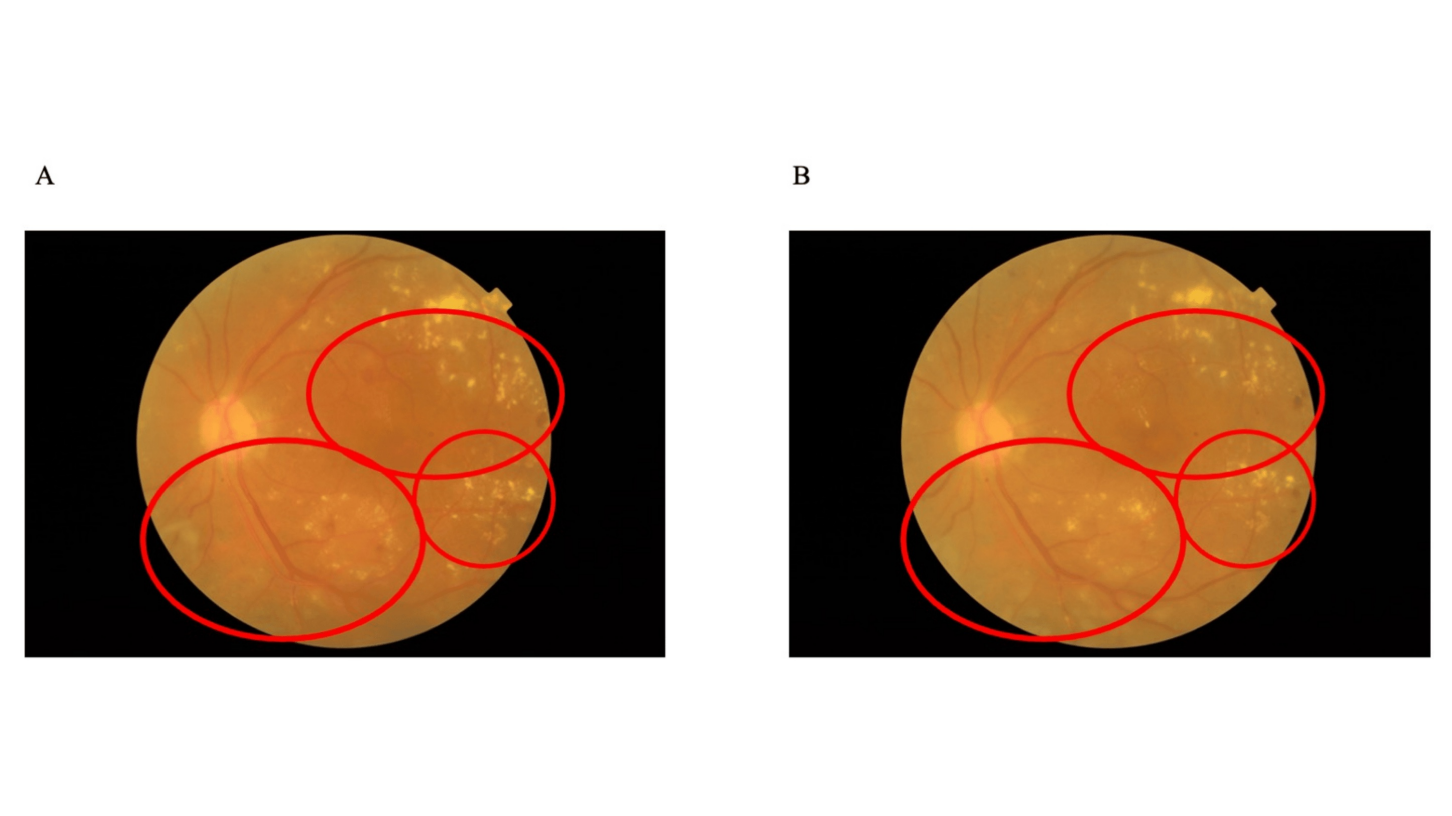
Left fundus findings at (A) admission and (B) after home follow-up There was no new hemorrhage during the intervention period

There were five passive SS sessions during the hospitalization period and 15 active SS sessions during the home follow-up period. The average daily number of steps (mean ± standard deviation) was 17,217 ± 3,106 and 15,211 ± 4,894 steps/day in the hospitalization and home follow-up period, respectively. No new intraocular hemorrhage or exacerbations of vitreous hemorrhage occurred during the intervention period (Figure 4). The IOP (right/left) measurements are as follows: 11/14 mmHg on admission, 11/12 mmHg on day 6 of admission, 12/11 mmHg on discharge, and 12/14 mmHg after home follow-up, with no marked variations.

The biochemical parameters are listed in Table 1. HbA1c level improved from 13.1% to 9.7% after one month.

**Table 1:** Biochemical parameters on admission and at the end of home follow-up. eGFR, estimated glomerular filtration rate; LDL, low-density lipoprotein; HDL, high-density lipoprotein

Biochemical parameters	Upon admission	After home follow-up	Reference range
HbA1c (%)	13.1	9.7	4.6–6.2
eGFR (mL/min/1.73m^2^)	33	33	≥60
HDL cholesterol (mg/dL)	85	88	38–90
LDL cholesterol (mg/dL)	170	141	65–163
Total cholesterol (mg/dL)	336	275	142–248
Triglyceride (mg/dL)	430	232	40–234

CAVI (right/left) was 8.3/8.5 at discharge (change from admission: -9.8%/-3.4%) and 7.8/8 after home follow-up (change from discharge: -6.0%/-6.1%), showing continued improvement. The ABI (right/left) was 0.78/0.94 at discharge, with only the left-side index showing a slight trend toward improvement, but no improvement was noted after home follow-up (Table 2).

**Table 2:** Arteriosclerosis-related indices at admission, at discharge, and after home follow-up CAVI, cardio-ankle vascular index; ABI, ankle-brachial index

Arteriosclerosis-related indices	Upon admission	Upon discharge	After home follow-up	Reference range
Right CAVI	9.2	8.3	7.8	<9.0
Left CAVI	8.8	8.5	8	
Right ABI	0.77	0.78	0.76	0.9–1.4
Left ABI	0.88	0.94	0.87	

**Figure 5:**
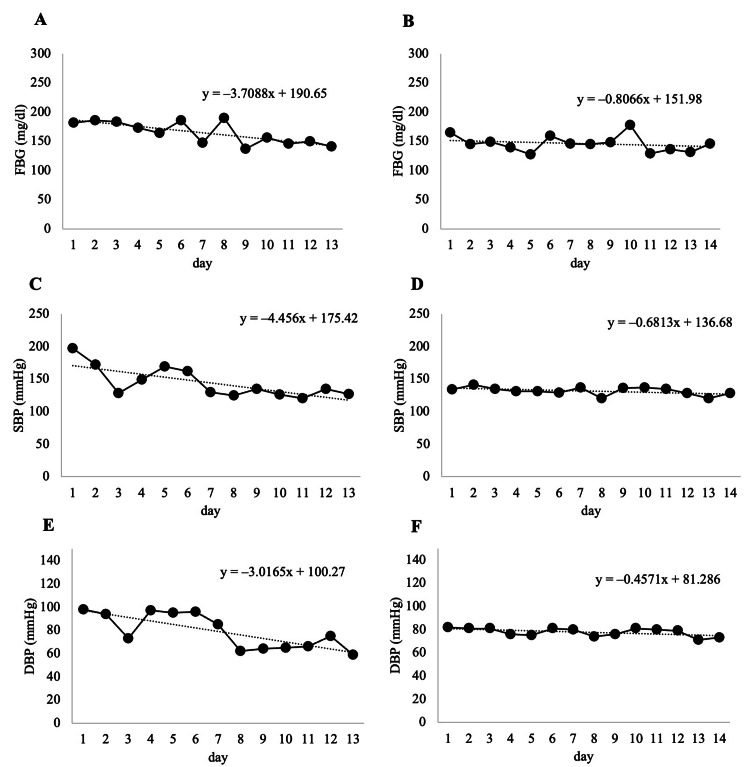
Fasting blood glucose level and blood pressure variation and regression lines during the hospitalization period and home follow-up period (A, C, E) Hospitalization period. (B, D, F) Home follow-up period. FBG, fasting blood glucose level; SBP, systolic blood pressure; DBP, diastolic blood pressure

The FBG, SBP, and DBP measurements are shown in Figure 5. The slopes of the regression lines in the hospitalization period and home follow-up period were -3.7 and -0.8 for FBG, -4.5 and -0.6 for SBP, and -3.0 and -0.5 for DBP. Both the parameters improved markedly during hospitalization. The improvement in the parameters was maintained after the home follow-up period, and a slight improvement in the trend was observed until after the home follow-up period.

## Discussion

This case report investigates the efficacy and safety of SS and walking at least 10,000 steps/day in patients with T2DM with PDR and PAD. Significant improvements in the levels of blood pressure, HbA1c, FBG, and CAVI were observed during the intervention period. No new fundus hemorrhages or changes in IOP were observed. These results demonstrate the safety and effectiveness of SS and walking at least 10,000 steps/day. Recent studies in healthy patients and patients with glaucoma have shown that aerobic exercise does not increase IOP [16,17]. However, there have been no reports on patients with PDR. In PDR presenting with vitreous hemorrhage, exercise therapy should not exacerbate bleeding. In this case, there was no new hemorrhage during the intervention period and no change in IOP. The patient’s blood pressure improved. This may have been owing to the selection of low-intensity exercises (approximately 2.5-3 METs [14] such as SS and daily walking).

In addition to exercise therapy, dietary recommendations were strictly followed. Therefore, the improvement in glycemic control, blood pressure, and CAVI levels during the hospitalization period can be attributed to the synergistic effects of exercise and diet therapy. Calorie intake during the home period was similar to that during the hospitalization period. In addition, the trend in FBG, SBP, and DBP levels was steady or showed improvement despite the resumption of drinking during the home follow-up period. CAVI showed a good improvement rate in both periods. The results suggest that SS and daily walking with hospitalization and home follow-up are useful for blood glucose and blood pressure control and for preventing arteriosclerosis.

Good improvement in the CAVI was a key feature of this case. Regarding the effect of SS, a four-week SS program for middle-aged men conducted in a study by Nishiwaki et al. resulted in improved CAVI from 7.7 to 7.2 [18]. The patient in the current case report was treated over a period of one month with a total of 20 sessions: five sessions of passive SS during the hospitalization period and 15 sessions of active SS during the home period. The frequency and duration of SS sessions were similar to those reported in the study by Nishiwaki et al. [18], and we believe that SS contributed to the improvement in CAVI. Tanaka et al. showed that habitual aerobic exercisers have lesser arteriosclerosis than non-habitual exercisers and that arteriosclerosis reduces with aerobic intervention [19]. Both low- and moderate-intensity (15% and 30% of maximal heart rate) aerobic exercises performed 15-30 min/day, three times/week, for eight weeks in middle-aged and older patients significantly improved CAVI, with improvement rates ranging from 9% to 11% [20]. A similar CAVI improvement rate was achieved despite the fact that the intervention period in this case was approximately four weeks. These findings suggest that the combination of low-intensity exercise, SS, and daily walking is a promising means of safely preventing arteriosclerosis in the short term. SS and aerobic exercise are thought to promote nitric oxide (NO) production by increasing shear stress on vascular endothelial cells and increased blood flow, respectively [21,22]. Continuous exercise and SS also have angiogenic effects [23,24]. CAVI is reduced by collateral blood vessels formed owing to neovascularization [25]. The cornerstone of exercise therapy for PAD is the development of collateral blood vessels through walking exercises. In the present case report, the patient’s PAD was Fontaine class 1 and he did not present with gait disturbance; therefore, the number of steps for the daily walking routine could be set high. In other words, good CAVI improvement with the combination of SS and walking was owing to the synergistic effect of NO-induced vascular smooth muscle relaxation and angiogenesis. Improvement in CAVI may prevent coronary artery disease and the development of neck atherosclerosis [26], thereby reducing mortality [27] and having a positive impact on long-term life expectancy.

The limitations of this study include the fact that it is a single-patient case report and is a short-duration study of approximately four weeks. Therefore, it remains unclear whether this applies to all cases of PDR and PAD complications. IOP was difficult to measure during exercise. However, we were able to show that aggressive exercise intervention with controlled exercise intensity is safe and effective in some cases with serious complications. Future large-scale studies on the safety and efficacy of exercise therapy in patients with T2DM complicated with PDR and PAD are needed.

## Conclusions

In this case report, the safety and efficacy of SS and walking at least 10,000 steps/day for approximately four weeks were tested in a patient with T2DM presenting with PDR and PAD. The results showed no new intraocular hemorrhages or changes in IOP and improvements in blood pressure, glycemic control, and CAVI levels. This case report suggests that SS and walking more than 10,000 steps/day can be safely performed and contribute to improved glycemic control and improved arterial stiffness.
